# Ontological Security, Trauma and Violence, and the Protection of Women: Polygamy Among Minority Communities

**DOI:** 10.3389/fpsyg.2021.743478

**Published:** 2021-10-01

**Authors:** Ayelet Harel-Shalev, Rebecca Kook

**Affiliations:** ^1^Conflict Management & Resolution Program, Faculty of Humanities and Social Sciences, Ben-Gurion University of the Negev, Beer-Sheva, Israel; ^2^Department of Politics and Government, Faculty of Humanities and Social Sciences, Ben-Gurion University of the Negev, Beer-Sheva, Israel

**Keywords:** minority women, challenges, ontological security, protection of women, polygamy, Israel

## Abstract

In this article, we examine the special challenges posed by the practice of polygamy to minority women, focusing on the ways that the state and the women confront the related experiences of violence and trauma associated with this practice. Based on analysis of both policy and interviews with women, we demonstrate the tension between the different mechanisms adopted by the state as opposed to those adopted by the women themselves. We suggest that the concept of ontological security is valuable for a deeper understanding of the range of state motivations in cases related to minority women, violence, and the right for protection. Our case study is the Bedouin community in Israel. We explore the relationship between individual and state-level conceptions of violence and trauma and the complex relationship between these two. We examine state discourses of ontological security through a gendered lens, as frameworks of belonging and mechanisms of exclusion.

## Introduction

Central to the definition of a democratic state is its role as guardian of citizens’ rights. Indeed, the history of democracy is to a very large extent the history of the evolution of these rights. A growing component of these rights has been a commitment to protect women’s rights ever since the emergence of the feminist movement at the end of the 19th century. Thus, as hallmarks of political and economic development, contemporary democratic regimes – prompted by international organizations, such as the OECD and the UN – show commitments to the promotion and protection of equal rights for women and to the protection of women from violence (OECD, 2020; UN Women, 2020). Concomitantly, the discourse on women’s equality, rights, and protection is increasingly being complicated by the inclusion of discussions relating to immigration, minority cultures and cultural values, and cultural belonging (Kagan et al., 2019), thereby raising fundamental questions regarding the nature of gender equality and the role of the state in guaranteeing equality and security for women.

A timely case in point is offered by an analysis of the practice of polygamy among the Bedouin minority in the south of Israel and of the recent policy changes of the State of Israel toward this practice. Polygamy is a gender-neutral term for marriages with multiple spouses, regardless of the gender combination (in 78% of cultures, plural marriage is practiced as polygyny, that is, one man married to multiple women; Davis, 2010, p. 1966). While polygamy is acknowledged as a phenomenon which is potentially harmful and discriminatory, it is also widely acknowledged that the lived experiences of women in polygamous families differ widely from place to place, culture to culture, and society to society (Harel-Shalev, 2013; Cook and Kelly, 2006; Gross, 2008). This said, it is clear that polygamy is far more than a set of marital practices, with implications for members of the practicing communities varying according to the wider political, national, and cultural contexts within which it takes place. The disadvantages and modes of subordination experienced by members of polygamous communities intersect with varied and different hierarchies of power which may ultimately contribute toward embedded gender, national, or religious inequality. Polygamy is practiced in over 850 different communities the world over (Aburabia, 2011a). In many countries, polygamy is prohibited by law; in others, it is legal, in yet others it is illegal but tolerated, and globally, it is outlawed by international human rights laws. It is widely acknowledged by international agencies that polygamy constitutes a practice that is discriminatory and harmful to the women and children involved and poses many challenges on physical, economic, and psychological levels. In Israel, polygamy is illegal, but the practice is nonetheless widespread among the Bedouin minority community, and, up until recently, it has largely been tolerated by the State. Critics of the State’s lack of enforcement of the law prohibiting polygamy have accused it of supporting harmful practices in a minority that suffers marginalization and discrimination and hence of abusing the rights of minority women, while proponents of the State’s policy have defended it in the name of multiculturalism, cultural autonomy, and the protection of minority rights. Others have argued that Israel’s failure to implement the prohibition derives from political considerations and the desire to coopt the dominant conservative Bedouin leaders (Boulos, 2019).

A change in the attitude of the State toward polygamy occurred in 2017 with the establishment of an inter-ministerial task force to make recommendations for combatting the practice. With the publication of its wide-ranging report in 2018 (The Inter-Ministerial Committee Dealing with the Negative Consequences of Polygamy Concluding Report, 2018; hereinafter “The Inter-Ministerial Report”), references to polygamy began to permeate official political discourse and became a common reference point for discussions related to the Arab-Bedouin minority. This discourse included the need for the protection of Bedouin women from violence as part of wider discussions of identity, gender, and identification with the Israeli state.

In this article, we examine the special challenges posed by the practice of polygamy to minority women, focusing on the ways that the state and the women confront the related experiences of trauma and violence associated with this practice. Based on analysis of both policy papers and interviews with women, we demonstrate the tension between the different mechanisms adopted by the state as opposed to those adopted by the women themselves. Our methodology is based on narrative and content analysis. The article critically explores the role of the state in guaranteeing the protection of women and investigates the wider contexts in which states formulate notions of gender (in)security. We suggest that the concept of ontological security is central for a deeper understanding of the range of state motivations in cases related to minority women, violence, and the right for protection. We explore the relationship between individual and state-level conceptions of violence and trauma and the complex relationship between these two. In addition, we examine state discourses of ontological security through a gendered lens, as frameworks of belonging and mechanisms of exclusion.

We make three main arguments. The first focuses on the relationship between individual and state-level conceptions of ontological security and the complex interconnectedness between these two narratives of “self.” We propose that notions of ontological security play a central role both in the ways states formulate policies related to the protection of women and in the ways women narrate experiences of trauma and violence. We argue that the concept of ontological security may be used to promote our understanding of the role of the state in protecting women from trauma and violence by focusing on the particular interpretations of security that contextualize state policies in this regard. In our second argument, we suggest that policies aimed at protecting marginalized minority women cannot be fully understood without accounting for the relationship between ontological security and narratives of national belonging as they are promoted by the state (Stein, 2017). Finally, we argue that, in the context of multicultural politics, states often make strategic use of gendered narratives of ontological security, such as the narrative recently promoted by Israel with regard to the Bedouin practice of polygamy, to further entrench national narratives of belonging.

### Literature Review

#### Ontological (In)security – Narratives of Self and National Belonging

The use of the notion of “ontology” as part of a wider focus on the fundamental elements of existence and being has become increasingly widespread in social science research, leading to what many have called the “ontological turn” (Todd, 2016; Holbraad and Pedersen, 2017). Generally, the concept of “ontological security” is employed to refer to the needs of individuals for a consistent sense of “self,” as a necessary condition for leading stable and healthy lives, thus adding a non-physical component to the idea of security. One of the main scholars of ontological security, Jennifer Mitzen, defines the term as follows: “Ontological security refers to the need to experience oneself as a whole, continuous person in time – as being rather than constantly changing – in order to realize a sense of agency” (Mitzen, 2006b, p. 342). Psychiatrist RD Laing, one of the main originators of the concept, described the “ontologically secure agent” as someone who has “a sense of his presence in the world as a real, alive, whole, and, in a temporal sense, a continuous person,” while its absence “renders the ordinary circumstances of everyday life [as] constitute[ing] a continual and deadly threat” (as quoted in Zarakol, 2017, pp. 39–40, 50). In the sociological iteration of the term, emphasis is placed on the importance of routine forms of behavior and knowledge, known otherwise as habitualization or habitus (Berger and Luckmann, 1991). The promotion and maintenance of a coherent biography as a point of reference is an essential component of this routine. Thus, inherent in the idea of ontological security is the importance of providing for shared ontological structures that provide for a shared social context (Mitzen, 2006b).

Any analysis of ontological security, on the collective level, will thus draw attention to collective biographical narratives and to routinized practices as modes for its construction and sustainability, helping us to understand how such practices shape political possibilities and outcomes (Kinnvall, 2004). In the context of political science research, this perspective of ontological security suggests that state actors recognize the importance of ontological security in addition to physical security and act in ways that contribute to a stable sense of the collective “self” (e.g., Huysmans, 1998; Lang, 2002; Kinnvall, 2004, 2006, 2015; Zarakol, 2010; Rumelili, 2015; Subotić, 2016). It is, thus, currently commonplace to assume that states are ontological security-seeking agents (Mitzen, 2006a,b; Delehanty and Steele, 2009; Zarakol, 2017). From this perspective, the legitimacy of the state ultimately rests not only on its capacity to provide physical security, but on its capacity to provide *order* – not a particular content of order but the function of *ordering*, of making life not only possible and sustainable, but also intelligible (Huysmans, 1998).

States’ conceptions of self-identity, in terms of ontological security theory, are constructed internally through the development of autobiographical narratives that draw upon national histories and selective historical moments to provide “comforting stories [about the self] in times of increased ontological insecurity and existential anxiety” (Kinnvall, 2004, p. 755). Whether related to the existential effects of modernity and liberalization or the far-ranging implications of mass migrations and regional conflicts, the need for a secure and continual sense of self is promoted and offered by the state as a necessary component of its physical, social, and political integrity (Giddens, 1990; Huysmans, 2006; Yuval-Davis, 2011). The provision of ontological security by the state is often bolstered by the use of narratives that highlight the insecurity, dread, and anxiety that result from a lack of security. In general, as Kinnvall (2015, p. 518) has noted, the production of security is also the production of insecurity.

While the discursive construction of fear, anxiety, and threat is intertwined with a variety of factors and feelings related to processes of economic and political change, recent research suggests that over the past few decades, narratives of fear and anxiety are often tied into general feelings of shared cultural and/or national “loss” (Mudde, 2007). As reflected in the widespread rise of right-wing and populist parties (Norris, 2019), the promotion of narratives of insecurity that construct immigrants and multiculturalism as threats to identity appeal to the tendency of individuals to overcome their own insecurities by viewing minorities and immigrants as menacing the integrity of the national community and as obstacles to the maintenance of a continuous stable sense of collective self (Minkenberg and Pytlas, 2012).

#### Gendered Readings of Ontological (In)security

Scholars of feminist international relations have long paid attention to the different gendered assumptions and premises underlying state power (Tickner, 1996; Golan, 2015); to how centralized power is conceptually represented as embodying and representing “masculine” norms (True, 2003); and to how security studies have tended to devalue other aspects of “security,” such as global inequality or environmental issues that are not within the “masculinized” understanding of security that focuses on war and conflict (Tickner, 1996, pp. 48–49). This disciplinary perspective underscores the fact that the sense of self that is constructed through discourses and practices aimed at promoting security is constructed mainly as masculine – rendering women and their rights and protection as subordinate and marginal (Tickner, 1996; True, 2003).

Shifting from a focus on constructions of masculinity inherent to security studies, recent research has looked at the different ways the promotion of ontological security by the state contributes toward the construction of femininity and issues of women’s rights and protection (Kinnvall, 2004; Delehanty and Steele, 2009). Given that the provision of ontological security is connected to the knowledge that one’s most intimate community is protected and secure within the larger political, social, and economic landscape, formulations of ontological security have roots in gendered concepts of family, intimacy, and home – and in the type of belonging that we associate with “home” (Massey, 1992; Mitzen, 2018). These connections, which rhetorically link the centrality of intimacy and home to a sense of security, add additional components to the political construction of femininity and womanhood. As Kinnvall (2004, p. 761) has noted, “The nation [becomes] associated with home, the place where the door will always be open for you, where a fire will be lit upon arrival and where you will receive the warmth of your mother’s care. Essentialist views of women, men, femininity, and masculinity are at the basis of such family metaphors ….” Kinnvall (2004, 2015, 2019) has contributed to an understanding of the gendered relationship between nation, security, and home and how this gendered conception is linked to ideas of the nation-as-family. She builds on significant previous research examining the nationalist trope of the woman as the protector and progenitor of the nation and how this trope has been woven into nationalist strategies (Berkovitch, 1997; Yuval-Davis, 2011). However, as Balibar (1991) and others have pointed out, images of gender, race, and nation are never far apart (Kinnvall, 2004, p. 782). In this sense, family metaphors have served to assign “others” to the ranks of second-class citizens, with women and minorities often being denied any direct relation to national agency and women from minority groups sometimes being perceived as threatening the safety and stability of the nation (Rossdale, 2015; Gazit, 2020).

Finally, recent feminist scholarship on fertility policies has contributed to this perspective by expanding our understanding of the construction of women as progenitors of the nation and the way fertility policies engage with ontological-security narratives (Leuprecht, 2010; Stopler, 2011). As Turner (2001) noted, the state promotes the desirability of fertility and reproduction as a foundation of social participation, thereby underscoring the demographic objective of securing and sustaining the connection between reproduction and citizenship. Nevertheless, past and present experience has taught us that fertility policies set by states and communities to respond to their population’s needs have more often than not ignored the rights and needs of the women recruited to carry them through and have privileged the rights of majority women over those of minority women. This preferential treatment is especially discriminatory against women in minority cultural communities who suffer from multiple levels of subordination. In these contexts, fertility can be seen as a component of security – when focused on women members of the state majority (Skey, 2010) – or as a threat and a component of insecurity – when focused on women members of marginalized or subordinate minorities. The discrepancy between the needs and rights of women as formulated by state legislators and as articulated by women themselves reinforces the need to explore vernacular expressions of ontological security alongside state-promoted narratives (Vaughan-Williams and Stevens, 2016).

Thus, while significant research has looked at the gendered assumptions underlying discourses of ontological security, less attention has been focused on the propensity of states to employ narratives of ontological security when dealing with issues of the protection of women – particularly of minority women. The reality of minority Muslim women globally is a case in point in that they have been targeted both as an object of state protection and simultaneously as the embodiment of a threat to the state, thus serving a double role in discourses of ontological security. Moreover, the commitment of the west to protect Muslim women has been systematically narrated in terms taken from the discursive landscape of *ontological* security and less from that of physical security (Kinnvall, 2019). Thus, for example, certain Western states with high numbers of Muslim immigrants have passed legislation aimed at limiting, and in some instances prohibiting, Islamic gender practices, such as veiling, the maintenance of strong patriarchal authority within the family and, in extreme cases, genital mutilation, that are considered as discriminatory and harmful to women (Abu-Lughod, 1990). These legislative initiatives, which may be viewed as curtailing cultural rights, were justified as a part of the state’s role as the protector of women’s human rights but were narrated as part of the state’s provision of ontological security to women (Abu-Lughod, 1990). Similarly, feminist scholars have analyzed the ways in which the United States justified its military involvement in Afghanistan (Shepherd, 2006) and Iraq by claiming that it was aimed at *saving the women*. Playing on anxieties and fears is also evident in references to fertility and “Islamic birthrates” made by some Western countries in the context of their “battle against terror” (Goodwin et al., 2005). Studies of anti-terror campaigns in Europe have documented how Muslim women are at times portrayed as “demographic bombs” (Goodwin et al., 2005), with both popular and state-promoted discourses using terms such as “demographic takeover” to refer to the high birthrates among Muslim immigrants (Busher, 2013). In summary, states can promote policies that are aimed at reducing or limiting practices that are seen as harmful to women and hence at enhancing their ontological security, while at the same time promoting policies that play into collective fears and anxieties regarding Muslim immigrants and citizens, thus promoting a sense of ontological insecurity for the other – security and insecurity woven together.

### The Case Study: The Consequences of the Practice of Polygamy Among the Bedouin Minority in Israel

Israel is a state with a deeply divided society, comprised of multiple religious, national, and ethnic communities and with a power structure that is rooted in ethnonational divisions (Karayanni, 2018; Ariely, 2021). This ethnonational hierarchical structure is premised, primarily, on the identification of the primary political nation with the ethnoreligious Jewish nation. Although all Israeli citizens, regardless of nationality, ethnicity and religion, are entitled to fundamental civic rights, including the right to vote, the ethnonational hierarchy privileges the Jewish national majority over the Arab-Palestinian minority. This national dimension of the hierarchy is considered to be a central component of Israel’s identity, as is also true for other ethnonational states (Waxman and Peleg, 2020).

#### The Arab-Bedouin Minority

The Bedouin community in Israel constitutes a minority within a minority. As Muslim Arabs, they are part of the Arab-Palestinian minority of Israeli citizens, a group made up of 80% Muslims and 20% Christians and constituting a minority of 18% of the total population (Abu-Kaf et al., 2019). The Arab-Palestinian minority in Israel – being a religious, national, and linguistic minority (Arabic-speaking in a predominantly Hebrew-speaking society) – thus embodies three overlapping cleavage lines (Ghanem and Rouhana, 2001). The minority as a whole suffers from institutional discrimination and inequality that is expressed in many ways, including discrimination in land ownership and property rights, discrimination in government budget allocation, and inequality in employment opportunities (Ghanem and Rouhana, 2001; Haklai, 2011).

The Bedouin Arabs, numbering approximately 230,000 individuals, constitute 3.5% of the Israeli population (Abu-Kaf et al., 2019). They constitute a distinct cultural traditional community within the larger Muslim Arab-Palestinian minority, but are, on the whole, poorer and more marginalized (Weissblei, 2017). Most live in communities in the southern region of Israel known as the Negev (Nasasra, 2017). Most of the Bedouin municipalities fall within the lowest clusters of the socio-economic status index (Central Bureau of Statistics, 2019), with estimations that almost half of the Bedouin men are unemployed, and levels of women’s unemployment reaching 78%.

While there are shades of variation among the different Bedouin communities, Bedouin society is, on the whole, a patriarchal society that abides by the norms of tribal tradition (Allassad-Alhuzail, 2018). Since the 1970s, Bedouin women have been subjected to conflicting processes. On the one hand, the weakening of traditional norms has granted girls and women access to higher levels of education and more varied employment opportunities (Harel-Shalev et al., 2020). On the other hand, the ambivalent and partial incorporation of modern norms and practices has made room for the entrenchment of deep patriarchal control over women, which is manifested in the persistence of traditional practices, such as polygamy and honor killings (Aburabia, 2011a; Yazbak and Kozma, 2017).

#### Polygamy in the Bedouin Community

Since its establishment in 1948, the State of Israel has granted full religious autonomy to all its religious communities. The implications of this religious autonomy are that issues of personal status fall under the exclusive authority of religious courts, with the exceptions of permitted age of marriage and polygamy. Although polygamy is permissible under certain interpretations of Islam and Judaism, Israel chose to limit religious autonomy over this marital practice and to prohibit and criminalize it under the Israeli Penal Law (Aburabia, 2011a). Despite the legal prohibition, over the years, the government has systematically failed to enforce the law, leading to the situation that over the past decade, 20–40% of Bedouin marriages in the Negev are believed to be polygamous (The Inter-Ministerial Report, 2018; Boulos, 2019). The consequences of this practice for the Arab minority in general and for the Bedouin community in particular are far-reaching. Polygamy results in higher levels of poverty, lower educational attainment for school children and for women, and, as we demonstrate below, overall impact on mental health and stability. These outcomes are felt by the entire community and are not limited to the polygamous families themselves (Aburabia, 2011a). Within these polygamous families, the husband will set up separate households, each with two to four wives. It is difficult to quote precise statistics regarding the number of polygamous marriages in this community, since to avoid criminal sanctions, most polygamous marriages are conducted in private religious ceremonies that are not registered in official records (Abu al-Asal, 2010; Boulos, 2019). There are, however, some data showing that over the past three decades, there has been a consistent number of polygamous marriages in the Bedouin society in Israel, irrespective of age, education, or socio-economic status (Aburabia, 2017).

To fully grasp the impact of polygamous realities on Bedouin women, it is necessary to examine these realities within the wider context of Bedouin patriarchy. As noted above, Bedouin communities continue to be characterized by a deepening of the existent patriarchal control over women (Aburabia, 2011b, 2017). Women are expected to maintain customary feminine roles within the home, including primary if not exclusive responsibility for childcare, care of the household, and care of the husband. Moreover, the authority of the traditional extended family structure is expanding, and it continues to maintain authority and control over every aspect of women’s lives (Aburabia, 2011a; Richter-Devroe, 2016). In parallel, data indicate that domestic violence is on the rise (Yazbak and Kozma, 2017; Allassad-Alhuzail, 2018). For decades, the Israel government has proactively supported traditional and tribal leaders and reinforced patriarchal institutions that foster submission and obedience as a means to lowering the costs of controlling the local population (Hasan, 2002; Boulos, 2019). In this respect, the status of Bedouin women is strongly affected by state policy.

However, in the context of the Israeli case, arguments that support the toleration of traditional customs, such as polygamy, in the name of cultural autonomy (The Inter-Ministerial Report, 2018) have been confronted with mounting evidence as to the harmful and indeed violent ramifications of polygamy for the women themselves and their children. Studies conducted from both within and outside the Bedouin community have, for example, indicated higher rates of mental health issues in polygamous women compared with monogamous women, including a higher prevalence of low self-esteem and depression (Al-Krenawi and Slonim-Nevo, 2008; Shepard, 2013). Moreover, there is some research-based evidence that women in communities in which polygamy is prevalent tend to suffer from anxiety even when they are not personally involved in polygamous marriage, since they frequently worry about what would occur should their husbands decide to take a second wife (Al-Krenawi and Lev-Wiesel, 2002; Abu-Shareb, 2017).

## Materials and Methods

Our research is based on two main sources. To investigate government policy toward polygamy and the overall perception of ontological security from the state’s perspective, we based our study on content analysis of policy documents, public speeches, and media coverage. To investigate the perspective of the women themselves regarding trauma and violence within the context of polygamy, we conducted group interviews and analyzed previously published personal narratives of women in polygamous families.

### Content Analysis

Our investigation of government policy is based on a careful reading and content analysis of two main sets of documents. To perform the content analysis, we read through the documents multiple times, discussed them together, and identified common themes. We then organized the content accordingly (Bengtsson, 2016). The two sets of documents are as follows:

1. The first is the government report published in 2018 and the related Knesset discussion protocols. We focus mainly on this report as it was the first significant government report on the issue ever produced;
2. The second included print and digital media articles regarding the report which showcased government official positions. In Israel, there are several news media outlets. Based on the Internet searches that we conducted, it became apparent that there were two main media outlets that published items related to our research. These included Haaretz and Yisrael Hayom – both news outlets which boast a very high readership and that cater to different audiences. Both of these news outsets appear digitally and in print. In addition, it is important to note that these two reflect the spectrum of political positions in Israel – from left leaning to right leaning (Korn, 2021). These articles included government official’s public statements about the report in particular and polygamy among the Bedouin community in general. It is important to note that as we were investigating the positions voiced by the official government, the items we chose were those that reflected the position of the leading coalition at the time led by then Prime Minister Netanyahu. The items we chose to discuss are representative of the way the government position was represented.

In our analysis, we identified several main themes which came up in the 2018 report’s analysis and which invoked central theoretical concepts from the study of ontological security. Accordingly, after identifying the main themes in the government report (our main source), we then collected media coverage and leading government official’s public statements concerning the report and conducted narrative analysis based on these themes.

### Interviews

Our research of the women’s perspective is based on both group and individual interviews. During the last 4years, we conducted four group interviews with 32 women, in polygamous and non-polygamous relationships, asking them about their lives, about their relationships, and about the topics of divorce and polygamy. The setting of group interviews is seen as uniquely suitable for facilitating critical feminist epistemologies in that group interviews can promote diverse forms of expressions of women’s insights and the shared, co-construction of knowledge (Kook et al., 2019; Kaplan et al., 2020). The interviews were conducted with 32 women divided into four groups, each group including eight adult women, with an age range chosen to represent different generations – that is, from 21years of age to 60years old. The women were from both recognized and unrecognized villages in the south of Israel. The research population is, of course, not representative of all Bedouin women; nonetheless, the group interviews gave us important insights derived from the field and from the dynamics that emerged within the groups. We employed a research assistant who facilitated both the recruitment process by snowball technique (see Cohen and Arieli, 2011) and the moderating of the groups. The research assistant conducted the interviews in Arabic and Hebrew. She translated the Arabic language segments into Hebrew in the transcription. Interviews were semi-structured and included questions related to everyday life, gender relations, family, marriage, and divorce. At the start of each session, the interviewees were assured of confidentiality and were asked to sign an informed consent form. Each group interview lasted between 120 and 160min and was audio-taped and transcribed. In addition to our primary sources, in our analysis, we included secondary sources as well – narrative analysis of 23 personal interviews conducted by Insaf Abu Shareb, a human rights lawyer with years of experience working with women in polygamous relationships. The age range of the interviewees was between 21 and 70years of age. They were from both recognized and unrecognized villages in the south. The transcriptions of these interviews were included in a public report submitted to members of the Israeli parliament in 2017 (Abu-Shareb, 2017). This report constituted one of the sources that the Government Report of 2018 was based on, and therefore, we decided to include these interviews in our analysis as well.

Analysis of the interview materials was in keeping with feminist narrative research that seeks to uncover previously neglected or misunderstood worlds of experience. We used narrative analysis to process the material. Listening to the women’s narratives enabled us to gain a deeper understanding of the various implications of polygamy, in line with the notion that there are various narratives of knowledge among women (Ackerly et al., 2006). In narrative analysis, scholars typically direct their research to working *with* narrative and *on* narrative (Bamberg, 2012). In working first with narrative, knowledge is constructed in a bottom-up direction. In the second phase, on narrative, we analyzed the interviewees’ narratives, by paying special attention to the ways in which individuals conform to and confirm existing social and political norms and circumstances (Wibben, 2011).

### Ethical Approval

This research was approved by the Human Subjects Research Committee, Ben-Gurion University – approval number 1782-1.

## Results I

### Women’s Perspectives

Opinions regarding the practice of polygamy within practicing cultures frequently vary within societies and families, across age groups, and gender, even among and within those who practice it (Al-Krenawi and Slonim-Nevo, 2008). Furthermore, perspectives of polygamy have been documented as varying even among the respondents themselves (Al-Krenawi and Slonim-Nevo, 2008; Shepard, 2013). Interview-based research on women from polygamous Bedouin families has provided evidence of the prevalence of feelings of humiliation, fear of physical violence, a deep sense of existential insecurity and isolation, and uncertainty about their self-identity and belonging (Huss and Cwikel, 2008; Abu-Shareb, 2017; Hassan, 2019; Sa’ar, 2020). Moreover, research indicates significant prevalence of mental health issues in polygamous women compared to monogamous women, including higher prevalence of low self-esteem, somatization, depression, anxiety, and psychiatric disorder in women involved in polygamous marital relationships (Al-Krenawi and Slonim-Nevo, 2008; Shepard, 2013). While some studies suggest better treatment toward first wives and their children, there is evidence among the Bedouin community that suggests that even first wives and their children are often neglected by their husband, since the first marriage is often dictated by tribal custom, while the second might be a product of choice among the partners (Al-Krenawi and Graham 2006; Al-Krenawi and Slonim-Nevo, 2008; Aburabia, 2017). The second wives are also a victim to this practice since their rights are fewer than women in monogamous marriages (Aburabia, 2017), and apparently, they suffer from similar mental health problems. In fact, research indicates higher levels of potency and lower levels of wife abuse among monogamous as compared to both first and second polygamous wives (Al-Krenawi and Lev-Wiesel, 2002). Furthermore, women in societies in which polygamy is prevalent might suffer from anxiety even when they are not personally involved in a polygamous marriage, since the decision of husband to take a second wife is always present (Al-Krenawi and Lev-Wiesel, 2002; Abu-Shareb, 2017).

In many cases, the uncertainty regarding their sense of self is related to the fact that, for most women, exiting a polygamous marriage within the traditional Bedouin community is not a realistic option, given both the extremely negative social implications of divorce and the lack of support given to such women by their families and communities (Abu-Shareb, 2017; Harel-Shalev et al., 2020). Given the centrality of community and family in a coherent sense of self in Bedouin society, threats to this sense of self can have significant consequences, particularly for women (Aburabia, 2011a). Many Bedouin women in polygamous marriages experience existential insecurity: They have reported a general feeling of helplessness, particularly given their lack of trust in their community and the ability or desire of the state to provide assistance. This lack of trust is enhanced by their reality of dire poverty and their limited access to sources of support and funding. These women often shoulder the burden of providing for their families, while being regularly abused by their husbands, both emotionally and physically, in some cases, even being prevented from access to basic services of healthcare, employment, and education (Abu-Shareb, 2017).

The findings from our narratives correspond well with these observations and emphasize the harsh realities. We now bring a few examples that exemplify this. It is important to note, that in most cases, the women did not express objections to the custom *per se*. They emphasize, however, the deep ontological insecurity that surrounds them in this context.

Here is an example of a woman with 12 children, in a polygamous marriage, sharing her experiences (Abu-Shareb, 2017):

I married when I was 16, and it was fine. Then, after he decided he wants another wife, my life turn to a nightmare. He went to live with the second wife and rarely came to visit us. A year later he left us completely, no visits, no support, no nothing. We were divorced by the tribe with no official papers, so I couldn’t receive support from the state. 12 people [referring to her kids] with no income, no support, hunger and poverty. All the burden is on my shoulders all the sudden. I feel exhausted.

As is evidenced from this narrative, polygamous women experience extreme difficulty and express extreme frustration. In addition, they describe a deep sense of ontological insecurity, uncertainties about their future, and about their self-identity (Abu-Shareb, 2017):

I am married as a second wife. Before that I was married to a relative of my family, a violent man. I married very young. He was violent and unbearable. I had three kids with him. One day he hit me in front of many people outside the village. People called the police and an ambulance. Off course, my kids were taken away from me, although the court’s verdict ruled that I have the custody. I was transferred to a secret shelter after the recovery at the hospital.

After some years, I met a man who promised to protect me, he was married. I agreed to marry him. He is the father of three of my children. We were poor, after some time he went back to his first wife and left us. I am poor, I had nowhere to go since my family expelled me, I had to move in with my mother-in-law, I am afraid to go out of the house, since I will probably be killed.

We were living with my mother-in-law who receive a small stipend from social security. I can’t buy a closet so our cloths are in plastic bags. I am suffering since I cannot provide for my kids. I turn to legal aid to receive individual support, it was a very long battle, with ups and downs, at the end I got this. And I have some air. I can provide for my children now and make them happy. I will not stand helpless anymore.

The individual stories of the women were difficult and involved elements of fear, violence, poverty, and the sense that there is no one to turn to – doubts in their self-identity and anxiety about their status in their community. Most narratives indicate that when a state stipend is available, it is helpful; however, in most cases, this requires a long and expensive legal struggle. In other cases, state support goes to the husbands. These battles leave the women feeling abandoned (Abu-Shareb, 2017):

Nobody cares about me. I don’t have the energy and the courage to continue.

Another woman emphasized that even if the husband remains and is not violent, the pain is unbearable (Abu-Shareb, 2017):

I am a first wife, he later married two additional wives. I never felt comfortable in this marriage. Even before he married he threatened that he would remarry and it hurt me. After his 2nd marriage I suffered more. When he got married I felt like someone stabbed me in the chest, this pain is always there in particular when I rethink about these humiliating moments. I cried and even tried to kill myself. I was very angry but I didn’t think of divorce. There was no reason for his additional marriage but his family encouraged him. To my family it was an ordinary thing, they don’t care. We and the other wives are not treated equally … I demanded alimony and I got it from him, after a court verdict. He comes to visit us, but he is missing in the house in our routine. He is not what he used to be. I feel that I am lacking something; I feel that something is wrong with me. I nevertheless, moved on, with my kids; slowly, I became independent and responsible for my home.

As evidenced in this narrative, divorce in the context of Bedouin society is not an easily accessible option. Despite rising rates of divorce partially related to polygamy (Aburabia, 2017), being a divorcee continues to be a highly vulnerable status for women – one that often entails severe social and economic hardships that keep some women imprisoned in a highly subordinate relationship with the larger community (Harel-Shalev et al., 2020). As some of the interviewees claim during a group interview, we conducted:

If you get divorced, it is hell.

Others, in a different group interview, describe the option of divorce as a “black stain” – women describe divorced women as weak and limited, entailing severe limitations to appear un-chaperoned in the public sphere:

Their parents and the extended family do not allow divorced women to go out … It is a black stain of the family.

At the same time, some of the divorcees are forced into polygamous marriages since it is considered worse off being divorced than being in a polygamous relationship (also see Hassan, 2019; Sa’ar, 2020).

Most women do not get divorced and those who chose to leave polygamous relationships talk about being forced to leave their community and feeling abandoned (Abu-Shareb, 2017):

I was in polygamous marriage, a second wife. At first it was relatively fine, but then the violence started. My husband used to hit me all the time, even when I was pregnant …. I had to run away to a shelter, although it is unacceptable in our society. They took my kids away from me to their father. The whole process was run by men and they decided what would I do and how, I didn’t have a choice … I lost my kids.

These women often worry about their status in their community and their self-identity – they would rather not leave their community but at the same time they worry about their lives. Some women noted that polygamy is a part of their religion and tradition, and some even admitted that “if the man takes care of all his wives” then it might work. Others rejecting the idea altogether. All were very skeptical that the state could intervene and make their situation better.

We are aware that in some cases, women join polygamous marriages as a second or a third wife, in order to gain the status of a married women, to be “secure” and find shelter (Hassan, 2019; Sa’ar, 2020). However, in our research, the narratives by the interviewees, both among first wives and second wives in polygamous marriages, reflect several recurring themes. Overall, women in polygamous marriage are often presumably married but they need to take the burden of providing for their families by themselves, and they are regularly abused both emotionally and physically, and often are even prevented from gaining basic services of healthcare, employment, and education. Some of these women have no support whatsoever from their families and their husband’s family and are often neglected by the state as well. Their ontological insecurity is evident and striking they feel humiliated and often experience both emotional pain and physical pain. Many shared that they felt unaccepted by the community – they lost their status in their tribe. Nevertheless, in this deep sense of helplessness and ontological insecurity, many of them shared their efforts to overcome the obstacles.

## Results II

### The State’s Perspective

In Israel, a state founded within the geopolitical context of widespread opposition and resistance, issues of physical security have always been central to government policy (Barak and Sheffer, 2009). Objections of Arab states to Israel’s existence were, from the onset, not limited to its physical presence but also intertwined with opposition to its identity as a Jewish state and therefore to its *ontological identity*. Thus, over the years, Israel has sought not merely to guarantee its physical survival, but also to gain acceptance and recognition of its identity as a Jewish state and hence of the authority of its political institutions to provide the Jewish people with a concrete representation of their national identity and a secure ontological framework. Indeed, for many of the neighboring Arab states, as well as for the stateless Palestinian people, the centrality of Israel’s self-declared identity as a Jewish state has constituted a focus of opposition on the basis of its exclusionary attitude toward non-Jews, in general, and Arab-Palestinians, in particular (Barak and Sheffer, 2009). It is in this wider context of the centrality of identity in the Israeli-Palestinian and Israeli-Arab conflict that scholars have recently adopted the concept of ontological security to investigate the dynamics of the conflict and attempts at its resolution (Mitzen, 2006b; Rumelili, 2015; Subotić, 2016). Israel’s efforts to promote and maintain its identity as a Jewish state in the wider context of ontological security are, however, not limited to the international arena. On the contrary, the main policy efforts aimed at securing this identity take place within the domestic arena. Within the domestic arena, two discoursive elements have emerged as central to the discussion about identity: The first is the discourse of demography which links between the ability of Israel to maintain its identity as a Jewish state and the demographic balance between Jews and non-Jews, and the second is the discourse of modernization which links between Israel’s identity as a democratic state and its modernizing role in the larger Middle Eastern landscape.

One of the prominent policy acts of the Israel government in the domestic context was the passing, in 2018, of the Basic Law: Israel as Nation-State of the Jewish People, informally known – and referred to here – as the Nationality Bill. This law, legally enforcing Israel’s identity as the nation-state of the Jewish people, states that the right to national self-determination in the State of Israel is unique to the Jewish people, that Hebrew is the official language and that therefore implicitly that the heritage and history of the Jewish people is the state’s sole tradition. The Nationality Bill, promoted by Prime Minister Netanyahu, offers a narrative – story with meaning, a story about belonging, and a story about identity. The Law provoked a great deal of controversy within and beyond the country, although many noted that it merely entrenched in law what was already known (Lustig, 2020). Seen through the lens of ontological security, however, its purpose becomes clear. To the extent that narratives of ontological security are basically stories that states tell themselves and the international community about their own identity, the narrative promoted by the Nationality Bill tells a story about what Israel is and the conditions necessary for making Israel secure in this identity. By legislating the identity of the state, the Law can be seen as providing Israelis with a stable and secure framework that ensures the continuity and integrity of their national identity by explicitly defining who belongs and making clear the hierarchies of membership. Within this story, the non-Jewish, Arab minority communities have a tenuous and, some may say, insecure role.

#### Israeli Policy Toward Polygamy – Engaging With Ontological (In)security

The publication of the Inter-Ministerial Committee’s Report on polygamy, and the ensuing public campaign that accompanied it, preceded the passing of the Nationality Law by 1year. From the perspective of the proximity of the two events, we suggest that Israel’s campaign against polygamy can be read as a chapter in the master narrative of its ontological security, and in its spirit, a chapter that highlights its gendered elements. The “polygamy campaign” tells a story regarding the essence of Israel’s identity and about who belongs and who does not. Moreover, and most significantly for this article, the polygamy campaign presents gender identity as an explicit and central actor in this narrative. While gender is always implicitly present in security narratives, analysis of the polygamy campaign through the lens of ontological security exposes the ways gender informs our sense of individual and collective self.

As noted above, polygamy is illegal in Israel and constitutes a criminal offense under Israeli law. The prohibition dates back to the promulgation of the Women’s Equal Rights Law in 1951, which included a clause specifically declaring polygamy as illegal. Overall, this law has played a seminal role in establishing women’s formal equality, by determining the legal boundaries of state intervention in family matters and by imposing what were at the time normative notions of femininity, family values, and overall gender roles (Berkovitch, 1997; Aburabia, 2011a). However, as Aburabia (2019, p. 311) has noted, while the Women’s Equal Rights Law defined women’s citizenship, the “citizenship of Muslim women was not at the core of the debates and was indirectly defined as secondary and marginal to the main definition of Jewish women’s citizenship.” According to Aburabia (2019), the criminalization of polygamy needs to be understood within the wider context of the exclusion and marginalization of the Arab minority and the lack of recognition of their interests, identity, and rights.

The above notwithstanding, over the years, the legal prohibition against polygamy has not been enforced. By 2018, only a handful of polygamy cases had been brought before the courts, with few convictions (Ben Zikri, 2020a). Similarly, until recently, the issue of polygamy has received scant media, popular, or even academic attention. Until the 2000s, the little attention that was paid to polygamy was almost exclusively in the field of mental health, with numerous studies focusing on the challenges faced by women in polygamous realities. Data were (and still is) hard to come by, but the government’s seemingly indifferent attitude supports Aburabia’s claim regarding the significance of the wider context of marginalization suffered by the Arab-Palestinian minority. Other scholars have made the stronger claim that the lack of enforcement derives from the interest of the government in cooperating with conservative patriarchal forces (religious and tribal leaders) within the Palestinian community (Hasan, 2002; Boulos, 2019).

This attitude of neglect changed abruptly in 2017 when Ayelet Shaked, the then Minister of Justice and member of the right-wing party *Habayit HaYehudi*, declared “war on polygamy.” Under her sponsorship, the Israel Government established an inter-ministerial task force mandated to conduct a comprehensive study on polygamy (The Government of Israel, 2017). The task force met and consulted with academics, mayors of Bedouin localities, Qadis of Sharia courts in Israel, members of Parliament, feminist activists, members of the police force, professionals from various ministries, social workers, and other professionals. In addition, it conducted visits to various Bedouin localities and institutions. The Final Report published by the task force in July 2018 clearly acknowledged that the State had failed to enforce the criminal prohibition on polygamy (The Inter-Ministerial Report, 2018; Boulos, 2019). Alongside the need for educational, employment, development, welfare and economic training programs, and measures, the Report also emphasized the need to enhance police and legal enforcement of the law (The Inter-Ministerial Report, 2018, pp. 215–229).

The publication of the Report drew extensive media attention and provoked official and unofficial government statements, including those of the Prime Minister, Benjamin Netanyahu (Netanyahu, 2018). The fight against polygamy thus became part of the national agenda. However, despite the wide scope and inter-disciplinary nature of the Report and its recommendations, media coverage and government briefings focused on very specific aspects of polygamy referred to in the Report, namely, the links between demography, fertility, and security and the links between the political authority of the state, the lawlessness of Bedouin society, and Israel’s role in “modernizing” them. These are the same issues which, as noted above, are central to Israel’s discourse of ontological security. It is thus not surprising that the publication of the report evoked much criticism from Bedouin feminist activists and scholars (Sa’ar, 2020).

#### Demography – The Jewish “Self” Identity of the State

A dominant theme that emerged in the public discourse surrounding polygamy was that of “demography,” particularly, the impact of increased birthrates resulting from the practice of polygamy on the demographic identity of the State. In most cases, references to “demography” were linked to notions of security threats. Shortly following the Report’s publication, the then Minister for Homeland Security, Gilad Erdan, was quoted as saying:

Polygamy among the Bedouin, and in particular when we talk about a Palestinian woman, is a crazy and dangerous phenomenon that we are obligated to eliminate by any means available. Children who grow up here and who are influenced by Palestinian incitement can eventually be tools in the hands of terror organizations and threaten the demography and our governability the Negev and beyond. Not only is the despicable polygamy harmful to women and children but it is a real security threat (Ben Zikri and Breiner, 2018).

This quote, from a very high-ranking political leader, illustrates the explicit connection made between terror and polygamy, which is intertwined with the notion of fertility. A similar quote was posted on Prime Minister Netanyahu’s Facebook page: “Polygamy … is not a “social and economic phenomenon” – it is first and foremost illegal …. [It] is harmful to the status of women and in addition disrupts the demographic balance in Israel ….” (Netanyahu, 2018). A similar tone was adopted by member of parliament Bezalel Smotrich, belonging to the far-right party *Habayit HaYehudi*:

the birth rate among Israel’s Bedouin community “is a bomb that must be handled, …. If we don’t defuse it, it will blow up on us much stronger … they double themselves every 12years, and so it’s something that needs handling … the more you modernize them, the less children they have (Ben Zikri, 2020b).

#### Modernization – “Saving” Muslim Women

The above quote links directly to the second theme – modernization – which highlights the belief that Muslim Bedouin women are helpless victims, under threat from their own society and in need of the modernizing influence and power of the State of Israel. This theme frames Israel as a Western liberal state characterized by modern norms and values, a state with low “modern” birthrates, and a state that is capable of saving the less developed Islamic community by promoting these values.

This theme emerged in the discussion on the Report of the main causes of polygamy, which privilege the cultural over the political: Statements similar in essence to “… polygamy is connected to the social and patriarchal structure of traditional societies characterized by homogenous norms and values based on a tribal social structure” are presented in one part of the Report (2018, p. 14), despite the widespread acknowledgment, elsewhere in the Report, of the role played by economic and political neglect in the perpetuation of the practice of polygamy. When addressing the causes of polygamy, the Report highlights the essentialist narrative: “The origin of the phenomenon among the Bedouin is in the Koran ….” (The Inter-Ministerial Report, 2018, p. 14). In another place: “[despite recent processes of change] the change in the status of women is slow and their social behavior is anchored deep within tradition and convention” (p. 30). Similarly, the Report also relates explicitly to the modernizing influence of Israel on the Bedouin community, linking it to the pitfalls of Bedouin culture:

[changes within the Bedouin community] are a result of the interaction with Israeli society and with modernization, globalization and urbanization …. The efforts of the government to provide services, to improve governance and the establishment of legal government institutions as is acceptable in Western countries – have promoted modernization processes and integration of the Bedouin community within - Israeli society (p. 35).

Thus, while the Report acknowledges that political and economic contexts factor into the reality of polygamy, it also portrays polygamy as an essentially cultural and traditional phenomenon, an inherent component of Bedouin identity, which should be eliminated, but only with the aid of resources and budgets (pp. 31–37). The modernization trope was picked up by leading members of parliament:

…. The more we westernize them … the lower the birthrates will become … the more the Bedouin population will be “*managed*”; will live in proper towns, will be educated and will enter the work force (Smotrich quoted in Ben Zikri, 2020a).

In a related theme, the Report placed strong emphasis on the role played by language in the ability to fight polygamy: “Lack of Hebrew language proficiency causes the Bedouin society to be isolated and closed to the rest of Israeli society and has direct impact on the status of women …” (The Inter-Ministerial Report, 2018, p. 141). The lack of Hebrew language proficiency was thus linked to the overall weakness and lack of power and the restricted access of the women and their children to higher levels of social and economic achievement. This lack of language proficiency perpetuates the dependence of the women on their husbands, who tend to know the language better and to reinforce the isolation of the women. While the importance of Hebrew language skills is substantiated by studies, different multilinguistic and multicultural perspectives were not explored at all in the Report, just as consideration was not given to the importance of maintaining a community’s native cultural environment. Hebrew was presented as the language of the powerful and as a means to achieving modernity and shedding the bonds of tradition. Thus, the implication was that only through shedding these bonds would women be able to fully modernize and escape the subordination of patriarchy, implying that adherence by Bedouin women to traditional norms, to their native language, perpetuates patriarchal subordination, and constitutes in itself an unsafe act.

The overall attitude and cultural assumptions of the Committee reflected the liberal feminist premise of the need “to save brown women from brown men” (Spivak, 1993). Thus, the logic of the Committee echoes the “politics of savior” prevalent in the West that looks at Islamic women as needing to be saved. This kind of logic avoids political questions and presents itself almost as a humanitarian project that expresses feelings of empathy in its drive to help and save polygamous women. Feminist Bedouin organizations that insist on the centrality of political questions such as issues of land ownership and systematic political de-legitimization were therefore viewed with suspicion, as ungrateful, and as provocateurs who are themselves preventing these helpless women from receiving the help they deserve (Sa’ar, 2020). Thus, ultimately, the Islamic community is portrayed as constituting a threat both to its own women and children and to Israeli society at large. In the public and political discourse surrounding the Report, the Bedouin community emerges as traditional – even primitive at times – abusive toward its women and children, lawless, and vulnerable to terror-inspiring ideologies and interests and, as such, is viewed as a direct threat to the physical and ontological security of the State. At the same time, by positioning the fight against polygamy as a component of government policy, the narrative contributes toward the promotion of a notion of Israeli identity as Jewish and Western (concepts that are ideologically intertwined), hetero-normative, modern, law abiding, and peace seeking, and as a chapter in the larger ontological security narrative discussed above in the context the Nationality Bill. The current article focused on the empirical case of the Israeli society but is relevant beyond this specific case, since states uses similar mechanisms of exclusion in order to enhance what they see as ontological security.

## Discussion

The focus of this article was the special challenges posed by the practice of polygamy to minority women, focusing on the ways that the state and the women confront the related experiences of trauma and violence associated with this practice. We demonstrated the advantages of employing the concept of ontological (in)security to investigate these issues in a nuanced and complex fashion. Special attention was given to the relationship between ontological security and gender and the challenges posed to minority women by these securitized narratives. Our analysis of the discourse surrounding polygamy in Israel in the context of ontological security is applicable to other empirical cases and reveals three main theoretical insights. The first concerns the relationship between individual and state-level conceptions of ontological security and the complex relationship between these two narratives of “self.” This insight reflects the stark contrast that exists between the ways in which the narratives of the women themselves highlight the long-lasting and deeply traumatic impact of the practice of polygamy to their overall mental, economic and social health, and the strategic nature of the discourse of the state. Overall, in this article, we propose that notions of ontological security play a meaningful and central role both in the ways states articulate policies related to the protection of women and in the ways women narrate experiences of trauma and violence. The second addresses what can be inferred from narratives of ontological security regarding the frameworks of belonging as presented by the state and specifically how these narratives often serve as mechanisms of exclusion. The third contributes to our understanding of the central and often defining role of gender and gender relations in state narratives of ontological security.

### Individual Vs. State-Centric Perspectives of Ontological Security

One of the central issues in the discourse surrounding ontological security is the tension between individual and state-centric approaches. Since polygamy as a social, cultural, and political practice is perceived as constituting a threat to ontological security both by the individual women who are trapped in polygamous arrangements and by state officials, the case of Israel’s attitude toward polygamy in the Bedouin community provides an opportunity to investigate the dynamics of these two perspectives in a shared template.

Indeed, the discussion presented above demonstrates that polygamy, for a vast majority of the women involved, constitutes a significant breakdown in their sense of ontological security. Our discussions of the narratives of the women indicated that polygamous women experience a more or less constant sense of alienation, displacement, and rupture, lacking a focus of belonging and the ability to maintain a stable sense of self. The lives of polygamous women were narrated as violent and humiliating. Moreover, the local community that should provide integrity, certainty, and a sense of belonging failed to consistently provide support to the women and their children. Interviews conducted with women who themselves were not involved in polygamous practices revealed that the threat of polygamy manifests as a constant presence forcing them to put up with abuse for fear of losing their status as “single” wives. This practice leads to enormous challenges and often devastating circumstances in which the women are exposed to violence and trauma on a daily basis.

The sense of ontological insecurity associated with polygamy from the State’s perspective was vastly different. As noted above, the State narrated a three-pronged story of polygamy: As being a source of ontological insecurity because of the ways, it impacted the demographic balance of the population, as being linked to traditional, non-modern modes of behavior and normative values, and as being connected to terrorist ideologies. Hence, polygamy was framed as a threat that is both physical and ontological, namely, that through their “unbridled” fertility, polygamous women seriously upset the delicate demographic balance of the State, threaten the identity of the State as Jewish, and cast a shadow on the State’s identity as Western, modern, and enlightened.

Thus, the different threats to ontological security are embodied in different forms and types of “self”: While the ontological security “self” narrated by the women described above was threatened by the behavior of men, the “self” that was narrated by the State was threatened by the implicit behavior of the women as vessels of fertility and as progenitors of demographic threats and potential terrorists. This divide exemplifies how states employ gendered narratives of ontological (in)security in a strategic fashion, portraying their policies as a means of protection of women against violence, but in fact motivated by interests related to national identity and security.

### Narratives of Ontological Security and Hierarchies of Belonging

Our second contribution focuses on the state. To the extent that states are ontological security-seeking actors, the process whereby states define their ontological security needs becomes a particular way of defining a “sense of state distinctiveness” (Mitzen, 2006b). Moreover, the logic of ontological security leads us to acknowledge the inherent relationship between this “sense of state distinctiveness” and the ontological needs of individuals for a stable and coherent biographic identity. Thus, the perspective of ontological security adds an additional dimension to theoretical discussions of national identity building and to the discussions of state trauma. As Mitzen noted: “Grounding a nation’s needs for ontological security in terms of individual needs in this way suggests that state institutions are not just an aggregate of leaders’ decisions, but that states also project self-images to which its citizens will be attached in complicated ways.” (Mitzen, 2006b, p. 352).

This theoretical insight was validated by our empirical analysis. Through the narratives of government leaders and the government Report, polygamy is presented as a real and immediate threat to the fundamental components of Israeli identity and hence as a threat to the existence of a stable cognitive environment. Polygamy is constructed as a threat to the stable continuity of the Jewish identity of the state, and hence to what is considered natural, appropriate, and acceptable to of Israel’s Jewish majority. By defining ontological security in these terms, the State enforces the distinction between the majority and the minority – between those who belong naturally and those who threaten the natural bonds of membership. By narrating polygamy as a threat to the demographic balance, the State is reinforcing the importance of a demographic balance to its stable identity, while also reinforcing hierarchies of belonging. Moreover, the modernization trope that identifies traditional practices as dangerous reinforces the imagination of the Jewish nation as besieged by the indigenous landscape but at the same time essential to its transformation. Insofar as Israel’s sense of self is embedded in these two tropes – besiegement and modernization – Israel’s insistence on highlighting the demographic dimensions of polygamy alongside its modernizing role of a traditional minorities can be seen, according to Zarakol (2010) as a “performance” of ontological (in)security and a reproduction of the state’s sense of “self.” Through the narrative of polygamy, the transformative capacity of the Jewish Israeli nation is reinforced and reconstituted in basic, binary formulations: modern vs. traditional; West vs. East; and rationality vs. passion. It is in the last of these binary constructions, which positions the rational capacity of the Israeli nation-state against the so-called sexualized passion of the Bedouin minority, that the gendered nature of the narrative is most apparent.

### The Gendered Nature of Ontological Security

With its focus on issues related to fertility, demography, and the home, gendered understandings of security are central to the rhetoric of ontological security. As Kinnvall has articulated, the need for a stable and continually available and welcoming “home” is at the root of our needs for security and is always present in state-level expressions of ontological security needs (Kinnvall, 2019). Women, as guardians of the home and as protectors of the family, will therefore always implicitly figure in these narratives. As the commitment to protect women’s rights and to prevent violence against women figures more and more centrally in discourses of state security, these gendered notions are moving from the background into the foreground.

Within the public discourse on polygamy, the role of the state as the protector of women’s physical integrity is deeply embedded within images and narratives of ontological security. The polygamous woman embodies the duality inherent in these narratives and images: She is at one and the same time the object of protection and also the source of a threat. Thus, through focus on the female body, the ontological security discourse constructs the hierarchical relationship between the Jewish majority and the non-Jewish Bedouin minority as one in which the majority is cast in the role of protector and simultaneously in the role of victim – protector of the physical and ontological security of Bedouin women, but victim of the practices in which they are implicated, namely potential terror, potential demographic takeover, and potential lawlessness (inherent in the concept of polygamy). Finally, within this narrative, gender is employed both as a central organizing trope and also as a strategic political mechanism. The need to “domesticate” minorities by eradicating patriarchal abusive practices is used not only as a trope but also as a strategic political mechanism.

In this article, we demonstrated that policies promoted by states to protect minority women from violence rely significantly on notions, concepts, and values related to ontological security, narratives of self and belonging, and identity. Thus, we suggest that the concept of ontological security should be seen as an organizing trope for a deeper understanding of the range of state motivations in cases related to women, violence, and the demand for security.

## Data Availability Statement

The original contributions presented in the study are included in the article/supplementary material, further inquiries can be directed to the corresponding author.

## Ethics Statement

The studies involving human participants were reviewed and approved by Ben Gurion University Ethics Committee. The patients/participants provided their written informed consent to participate in this study.

## Author Contributions

All authors listed have made a substantial, direct and intellectual contribution to the work, and approved it for publication.

## Funding

This research was funded by the Israel Science Foundation grant number 134/19 as well as by the Sol Leshin Grant for cooperation between UCLA and Ben Gurion University.

## Conflict of Interest

The authors declare that the research was conducted in the absence of any commercial or financial relationships that could be construed as a potential conflict of interest.

## Publisher’s Note

All claims expressed in this article are solely those of the authors and do not necessarily represent those of their affiliated organizations, or those of the publisher, the editors and the reviewers. Any product that may be evaluated in this article, or claim that may be made by its manufacturer, is not guaranteed or endorsed by the publisher.
